# Correction for Manohar et al., "A Multiwell-Plate *Caenorhabditis elegans* Assay for Assessing the Therapeutic Potential of Bacteriophages against Clinical Pathogens"

**DOI:** 10.1128/spectrum.02606-24

**Published:** 2024-11-11

**Authors:** Prasanth Manohar, Belinda Loh, Namasivayam Elangovan, Archana Loganathan, Ramesh Nachimuthu, Sebastian Leptihn

## AUTHOR CORRECTION

Volume 10, no. 1, e01393-21, 2022, https://doi.org/10.1128/spectrum.01393-21. Page 1, Affiliations: The fourth affiliation should read “Antibiotic Resistance and Phage Therapy Laboratory, School of Bio Sciences and Technology, Vellore Institute of Technology, Vellore, Tamil Nadu, India.”

