# Supplementary material for: A Multiwell-Plate Caenorhabditis elegans Assay for Assessing the Therapeutic Potential of Bacteriophages against Clinical Pathogens
Source: Microbiol Spectr. 2022 Feb 16;10(1):e01393-21. doi: 10.1128/spectrum.01393-21 (PMC8849058; doi:10.1128/spectrum.01393-21)
Supplement: SUPPLEMENTAL FILE 1 — Supplemental material. Download SPECTRUM01393-21_Supp_1_seq9.pdf, PDF file, 0.2 MB [file spectrum01393-21_supp_1_seq9.pdf]

Supporting Information

A multiwell-plate *Caenorhabditis elegans* assay for assessing the therapeutic potential of Bacteriophages against Clinical Pathogens

Prasanth Manohar[1,2], Belinda Loh[1], Namasivayam Elangovan[3], Archana Loganathan[4], Ramesh Nachimuthu*[4], Sebastian Leptihn*[1,5,6]

[1]Zhejiang University-University of Edinburgh (ZJE) Institute, Zhejiang University, School of Medicine, Haining, Zhejiang, PR China
[2]The Second Affiliated Hospital Zhejiang University (SAHZU), School of Medicine, Hangzhou, Zhejiang, PR China
[3]Department of Biotechnology, School of Bioscience, Periyar University, Salem, Tamil Nadu, India
[4]Antibiotic Resistance and Phage Therapy Lab, Department of Biomedical Science, School of Biosciences and Technology, Vellore, Tamil Nadu, India
[5]Department of Infectious Diseases, Sir Run Run Shaw Hospital, Zhejiang University, School of Medicine, Hangzhou, PR China
[6]University of Edinburgh Medical School, Biomedical Sciences, College of Medicine & Veterinary Medicine, The University of Edinburgh, 1 George Square, Edinburgh, EH8 9JZ, United Kingdom

***Corresponding authors:**

1. Prof. Dr Ramesh Nachimuthu
Assistant Professor,
School of Biosciences and Technology,
Vellore, Tamil Nadu, India
E-mail: drpnramesh@gmail.com

2. Prof. Dr Sebastian Leptihn
Associate Professor,
Zhejiang University-University of Edinburgh (ZJE) Institute,
Zhejiang University, School of Medicine,
Haining, Zhejiang, PR China
E-mail: Leptihn@intl.zju.edu.cn

Supplementary Figure 1

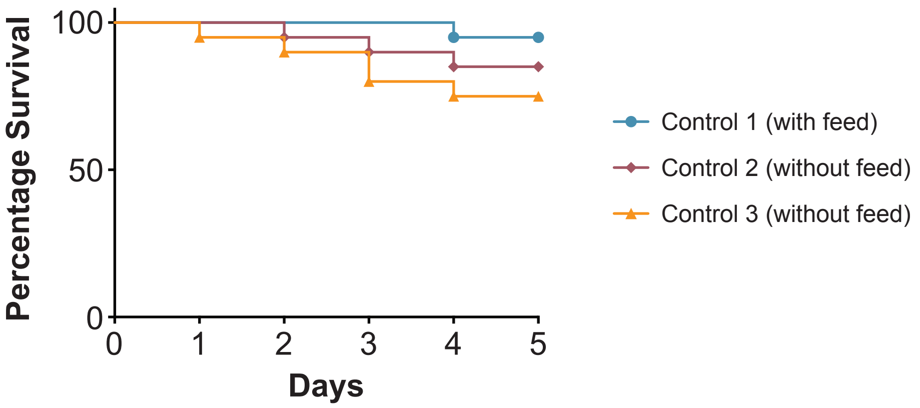

Supplementary Figure 1: Survival curves of *C. elegans*. Nematodes were kept in varying growth conditions; Control 1: nematodes were fed with *E. coli* OP50, Control 2 (liquid media) & Control 3 (solid-media): nematodes were not fed. Survival curves were plotted using Kaplan-Meier method and log-rank test was used to analyze the difference in survival rates in GraphPad Prism 7.0.

Supplementary Figure 2

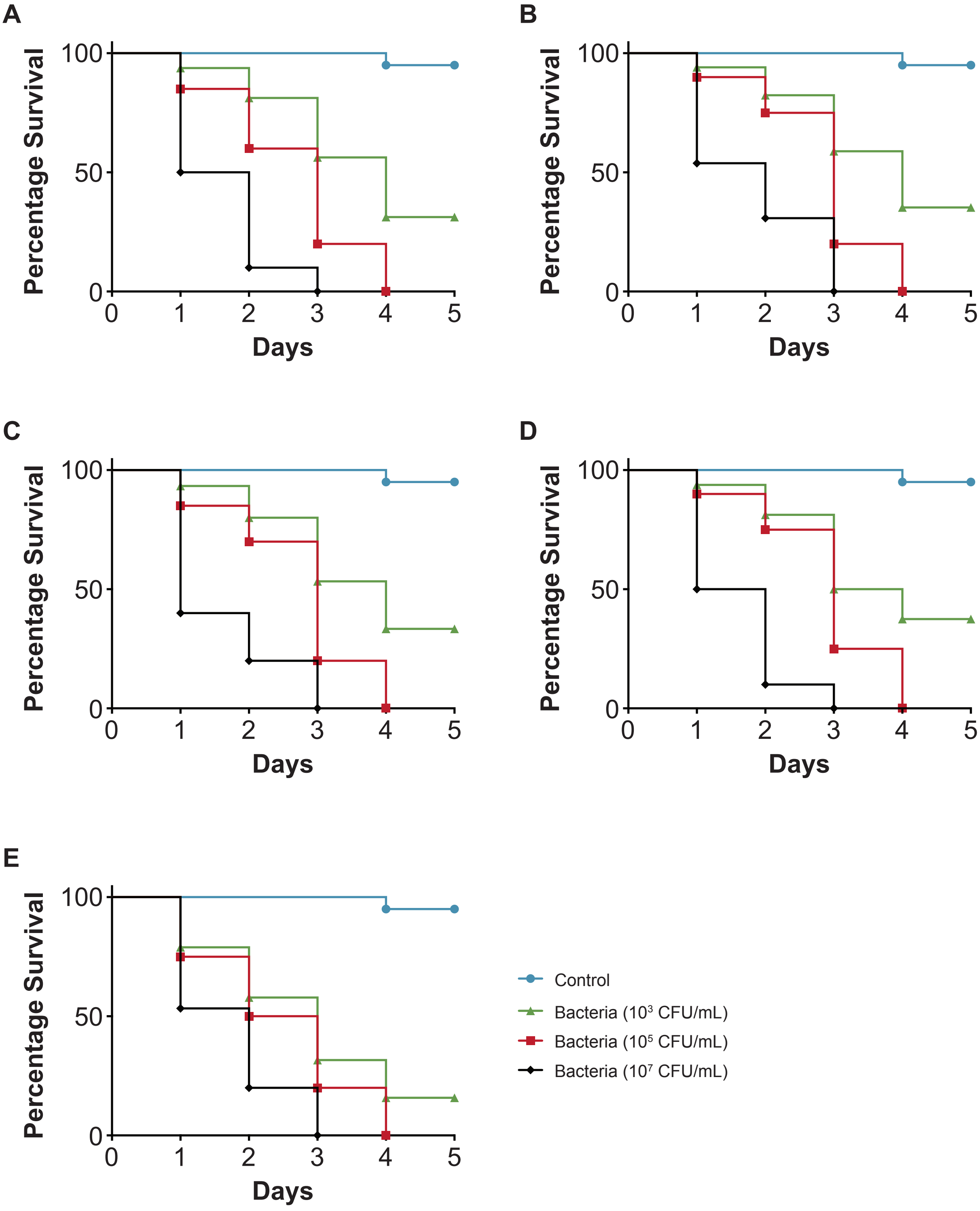

Supplementary Figure 2: Survival curves of nematodes (*C. elegans*) when infected with pathogenic bacteria at different concentrations, 10^3, 10^5 and 10^7 CFU/mL. (A) *C. elegans* infected with *Escherichia coli* 131; (B) *C. elegans* infected with *Escherichia coli* 311; (C) *C. elegans* infected with *Klebsiella pneumoniae* 235; (D) *C. elegans* infected with *Enterobacter cloacae* 140. The survival curves were plotted using Kaplan-Meier method in GraphPad Prism 7.0.

Supplementary Figure 3

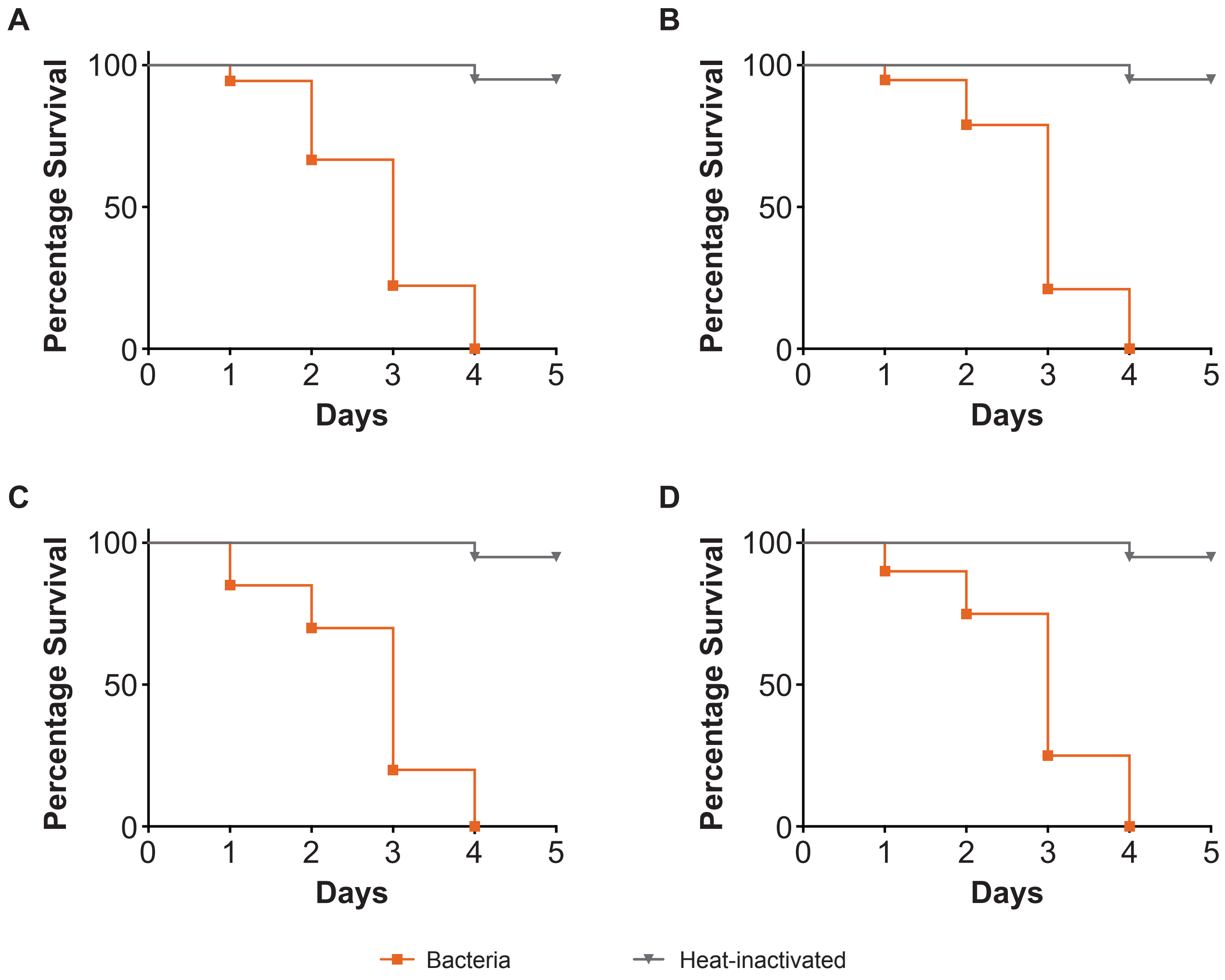

Supplementary Figure 3: Survival curves of nematodes (*C. elegans*) when infected with live bacteria and heat-killed bacteria. (A) *C. elegans* infected with *Escherichia coli* 131; (B) *C. elegans* infected with *Escherichia coli* 311; (C) *C. elegans* infected with *Klebsiella pneumoniae* 235; (D) *C. elegans* infected with *Enterobacter cloacae* 140. The survival curves were plotted using Kaplan-Meier method in GraphPad Prism 7.0.
